# Genomic analysis of *Enterococcus durans* LAB18S, a potential probiotic strain isolated from cheese

**DOI:** 10.1590/1678-4685-GMB-2021-0201

**Published:** 2022-02-25

**Authors:** Carolina Baldisserotto Comerlato, Janira Prichula, Franciele Maboni Siqueira, Ana Carolina Ritter, Ana Paula Muterle Varela, Fabiana Quoos Mayer, Adriano Brandelli

**Affiliations:** 1 Universidade Federal do Rio Grande do Sul, Departamento de Ciência de Alimentos, Laboratório de Bioquímica e Microbiologia Aplicada, Porto Alegre, RS, Brazil.; 2 Universidade Federal de Ciências da Saúde de Porto Alegre, Laboratório de cocos Gram-positivos, Porto Alegre, RS, Brazil.; 3 Universidade Federal do Rio Grande do Sul, Departamento de Patologia Clínica Veterinária, Laboratório de Bacteriologia Clínica, Porto Alegre, RS, Brazil.; 4 Secretaria da Agricultura Pecuária e Irrigação do RS, Departamento de Diagnóstico e Pesquisa Agropecuária, Laboratório de Biologia Molecular, Eldorado do Sul, RS, Brazil.

**Keywords:** Enterococcus, probiotc, prebiotic, genome, selenoproteins

## Abstract

Gut microbiota exerts a fundamental role in human health and increased evidence supports the beneficial role of probiotic microorganisms in the maintenance of intestinal health. *Enterococcus durans* LAB18S was previously isolated from soft cheese and showed some desirable *in vitro* probiotic properties, for that reason its genome was sequenced and evaluated for genes that can be relevant for probiotic activity and are involved in selenium metabolism. Genome sequencing was performed using the Illumina MiSeq System. A variety of genes potentially associated with probiotic properties, including adhesion capability, viability at low pH, bile salt resistance, antimicrobial activity, and utilization of prebiotic fructooligosaccharides (FOS) were identified. The strain showed tolerance to acid pH and bile salts, exhibited antimicrobial activity and thrived on prebiotic oligosaccharides. Six genes involved in selenium metabolism were predicted. Analysis of the SECIS element showed twelve known selenoprotein candidates. *E. durans* LAB18S was the only food isolate showing absence of plasmids, virulence and antimicrobial resistance genes, when compared with other 30 *E. durans* genomes. The results of this study provide evidence supporting the potential of *E. durans* LAB18S as alternative for probiotic formulations.

## Introduction

*Enterococcus* genus belongs to the group of lactic acid bacteria (LAB) of the phylum Firmicutes, showing the ability to survive under various environmental conditions (Byappanahalli et al., 2012). This genus is an important component of the intestinal microbiota of humans and other animals and is found in commercial products, such as Cernivet® and FortiFlora® (containing *Enterococcus faecium* SF68®, Cerbios -Pharma SA, Switzerland) and Symbioflor® 1 with *Enterococcus faecalis* (Symbiopharm, Herborn, Germany) (Hanchi et al., 2018). Many enterococci isolated from fermented dairy products proven to be natural probiotics and have been considered beneficial and safe to the host (Franz et al., 2011). 

Currently, the role of probiotic bacteria in gut health and functionality of human, livestock animals and pets has been greatly emphasized. The intestinal microbiome has a great importance in human health, promoting intestinal homeostasis, development of the immune system, protection against pathogens and stimulating the production of micronutrients and energy (Clemente et al., 2012; Martín and Langella, 2019).

Some *in vitro* assays are recommended to characterize a microorganism with probiotic potential, including adherence to human and/or mucosal epithelial cells, antimicrobial activity against pathogens, ability to decrease the adhesion of pathogens and stimulate the hydrolysis of bile salts (Hill *et al*., 2014). These assays have become the dogma for probiotic characterization, but phenotypic characterization is not enough to provide a full description of probiotic microorganisms. Thus, the study of genomic data obtained by high-throughput DNA sequencing tools may provide novel useful information, expanding the current knowledge on probiotic strains. Genomic analysis may be useful to identify genes related to probiotic properties and to find additional molecules and metabolic routes that contribute to the specific activity of a probiotic strain (Li et al., 2018). These genes can codify proteins associated with survival to gastrointestinal tract transit, such as bile salt hydrolases, production of antimicrobial substances like bacteriocins, and beneficial enzymes, such as β-galactosidase (BGL) and inulinase (Ladero et al., 2013; Bonacina et al., 2017).

In addition to these probiotic characteristics, antioxidant properties play an important role and can be associated to the ability of a probiotic to produce selenoproteins. Selenium (Se) is a trace element known primarily for its functions in redox homeostasis as a promising chemo-preventive agent for cancer (Hartfield *et al*., 2006) and because it has beneficial effects associated with probiotic bacteria (Galano et al., 2013). The major biological form of Se is selenocysteine (Sec, the 21^st^ amino acid), which is co-translationally inserted into selenoproteins by recoding the UGA codon (Hatfield and Gladyshev, 2002). In bacteria, the mechanism of Sec biosynthesis and its insertion into proteins requires an in-frame UGA codon, a Sec insertion sequence element (SECIS). SECIS is a hairpin structure within the selenoprotein mRNA immediately downstream of the Sec codon encoding the UGA codon (Zhang and Gladyshev, 2005).

Although genome sequences of *Enterococcus* species like *E. faecalis* and *E. faecium* have been largely described (Bonacina et al., 2017; Zhong et al., 2017), minor information is available for *E. durans* (Li et al., 2018). The *E. durans* LAB18S was previously isolated from a typical Brazilian soft cheese and exhibited some desirable probiotic properties *in vitro* (Pieniz et al., 2015). In addition, this strain thrives in selenium enriched medium, accumulating this element in the biomass (Pieniz *et al*., 2017). Further research is needed to prove its potential health benefits and application as a probiotic lineage in the industry. Thus, the aim of this study was to characterize the genome of *E. durans* LAB18S strain, searching for relevant genes associated with probiotic properties and selenoproteins, in addition to performing comparative analyzes with *E. durans* genomes from different isolation sites.

## Material and Methods

### Genomic DNA preparation and high-throughput sequencing

*E. durans* LAB18S was isolated from soft cheese, was retrieved from the collection of Laboratory of Applied Microbiology and Biochemistry (Universidade Federal do Rio Grande do Sul, Porto Alegre, Brazil). The strain was maintained as frozen stock cultures in Brain Heart Infusion (BHI, Oxoid) containing 20% (v/v) glycerol. The bacterium was grown in MRS broth (de Man et al., 1960) at 37 °C at mid log phase (8 h).

*E. durans* LAB18S total DNA was extracted with phenol-chloroform following usual procedures and purified using a Genomic DNA Clean & Concentrator (Zymo Research). The quality and quantity of the DNA were assessed by spectrophotometry analysis using NanoDrop™ (Thermo Scientific) and fluorometry (Qubit™; Invitrogen), respectively. DNA fragment libraries were further prepared with 50 ng of DNA using a Nextera^TM^ XT DNA sample preparation kit and sequenced using an Illumina™ MiSeq System (2x250 paired-end reads with the Illumina^TM^ v2 reagent kit), manufacturer’s instructions. 

After quality checking with FastQC software, reads were trimmed with Geneious software (version 10.2.3) (https://www.geneious.com). The paired-end sequence reads were then assembled by *de novo* assembly using SPAdes 3.9.0 (Bankevich et al., 2012), and Geneious software version 10.2.3 followed by template-assisted assembly to the reference *E. durans* KLDS6.0933 (NZ_CP012366).

### Gene prediction and bioinformatics analysis

Annotation NCBI Prokaryotic Genome Annotation Pipeline (PGAAP) was employed to identify coding sequences (CDS) based on the best-placed reference protein set. Similarly, to aid the gene prediction and annotation, *E. durans* genome were performed by RAST (Rapid Annotation Subsystem Technology) webservice (https://rast.nmpdr.org). Genes of interest had their annotation refined manually. This Whole Genome Shotgun project has been deposited at DDBJ/ENA/GenBank under the accession NCVP00000000. The version described in this paper is version NCVP01000000.

Genes involved in the biosynthesis of secondary metabolites were analyzed *in silico* using the antiSMASH algorithm (Medema et al., 2011). We then used bSECISearch to predict candidates for bacterial SECIS elements and their putative coding genes with weight scores greater than the cutoff (> 30) in order to analyze the genome of *E. durans* LAB18S for full complement of selenoprotein genes (Zhang and Gladyshev, 2005). BLAST search (tblastn + blastx) was performed at NCBI to filter out false positive elements involved with selenium.

### Comparative analysis

Antimicrobial resistance genes were identified using ResFinder 3.2 (Zankari et al., 2012) following the thresholds 60% identity over a length of 60% coverage, respectively. VirulenceFinder (Joensen et al., 2014) and PlasmidFinder (Caratoli *et al*., 2014) were used to predict potential virulence genes and plasmids, respectively. Identification thresholds were set at 60% identity over a minimum length of 60% for PlasmidFinder, and 85% identity over a length of 60% for VirulenceFinder.

Core genome Single Nucleotide Polymorphism (SNP) tree were performed using Parsnp v1.2 program included in Harvest (Treangen et al., 2014). A total of 31 *E. durans* genomes, one draft genome from this study and 30 genomes from previous studies obtained from the NCBI database were used (Table 1). Core genome SNPs of *E. durans* were identified, the reference genome was randomly selected using the parameter ‘-r!’ and recombination regions were used (Treangen *et al*., 2014). An approximately maximum likelihood tree was constructed from concatenated SNPs using FastTree2 (Price et al., 2010), and interactive Tree Of Life (iTOL) v4 software (Letunik and Bork, 2019) were used for visualization and edition of the phylogenomic tree.

**Table 1 -:** Complementary information of *Enterococcus durans* genomes from NCBI.

Species	Strain	GenBank assembly	Genome size (bp)	Contigs	n50
*E. durans*	18S	GCF_003945985.1	2760363	61	210893
*E. durans*	4928STDY7071618	GCA_902162045.1	3173223	140	69586
*E. durans*	4928STDY7071587	GCA_902161685.1	3129748	140	53780
*E. durans*	4928STDY7071465	GCA_902160745.1	2952049	115	66799
*E. durans*	4928STDY7071468	GCA_902160735.1	3049809	33	266845
*E. durans*	4928STDY7071461	GCA_902160695.1	2843396	99	68904
*E. durans*	4928STDY7071424	GCA_902160425.1	2993992	134	66221
*E. durans*	4928STDY7071423	GCA_902160385.1	2987662	135	66543
*E. durans*	4928STDY7071358	GCA_902159875.1	2937777	113	70649
*E. durans*	4928STDY7071318	GCA_902159725.1	3070184	143	59610
*E. durans*	4928STDY7071647	GCA_902159525.1	2965835	49	151918
*E. durans*	4928STDY7071469	GCA_902159215.1	3065464	56	207797
*E. durans*	4928STDY7071427	GCA_902159205.1	2986615	134	63841
*E. durans*	4928STDY7071462	GCA_902159195.1	3052558	42	228325
*E. durans*	4928STDY7071385	GCA_902159095.1	3126814	142	53818
*E. durans*	NCTC8129	GCF_900447815.1	3259358	6	3126530
*E. durans*	NCTC8130	GCF_900447695.1	3357395	8	3078716
*E. durans*	OSY-EGY	GCF_004330425.1	3230625	227	52003
*E. durans*	am_0171	GCF_004167095.1	3002381	120	55320
*E. durans*	C11	GCF_004102865.1	2988164	115	54843
*E. durans*	P16CLA28	GCF_003796805.1	2886365	28	215484
*E. durans*	AF1132H	GCF_003465125.1	3083830	215	34708
*E. durans*	FDAARGOS_396	GCF_002554315.1	3395970	4	3104428
*E. durans*	BDGP3	GCA_002277935.1	2988928	2	2983334
*E. durans*	F0321E104	GCF_002077535.1	2931215	43	147275
*E. durans*	NBRC100479	GCF_001544215.1	3017302	122	53575
*E. durans*	IQ23	GCF_001455455.1	3125512	127	70907
*E. durans*	KLDS6.0930	GCF_001267865.1	3071879	3	2867090
*E. durans*	KLDS6.0933	GCF_001267395.1	3071804	3	2867028
*E. durans*	ATCC6056	GCF_000406985.1	3153755	19	411581
*E. durans*	IPLA655	GCF_000350465.1	3059052	145	73480

### Phenotypical characteristics

*E. durans* LAB18S was evaluated for tolerance to acid pH and bile salts, β-galactosidase activity and growth on prebiotic oligosaccharides.

*Acid tolerance*

The resistance under acid conditions was investigated according to Erkkila and Petaja (2000) with some modifications. *E. durans* LAB18S cells were grown in BHI (Brain Heat Infusion broth; Oxoid) without shaking at 37 °C for 24 h. Then, the culture was standardized at an optical density (OD_600_) = 1.0 ± 0.05. One milliliter of standardized culture was added into tubes containing 10 mL of sterile BHI broth with the following pH values: 2.0, 3.0, 4.0 and 7.0 (adjusted with HCl), in which pH 7.0 was used as a control. Viable cell counts were determined after exposure to acidic condition for 0, 1, 2, 3 and 4 h at 37 °C. The experiment was performed in triplicate. Survival cell counts were expressed as log values of colony-forming units per ml (CFU/mL). 

*Bile tolerance test*

Growth in the presence of 0.3% (w/v) oxbile was analyzed as described by Gilliland et al. (1984). Overnight grown (16 ± 2 h at 37 °C) assay cultures were centrifuged at 8,000 x g for 15 min at 4 °C and the pellet collected was resuspended in same volume of saline (0.85% NaCl). Fresh BHI broth (5 ml), without ox bile with pH 7 (for control), and BHI broth (5 ml) containing 2.5, 5, 10 and 15 mg/mL of ox bile was inoculated with 250 μl (5%) of cell suspension. The growth was monitored hourly by measuring the OD at 600 nm using spectrophotometer. The survival percentage was calculated as follows: % survival = final (OD) / control (OD) x 100.

*β-Galactosidase (BGL) activity*

BGL activity was assayed by a modified procedure, based on the method of Hang and Woodams (1994). The source of BGL was a cell-free supernatant of *E. durans* LAB18S culture in BHI broth (Brain Heart Infusion) and sonicated LAB18S cells. Besides, this isolate was grown in BHI broth supplemented with 10 g/L lactose and the same assay was performed. The reaction mixture (200 μL) contained 90 μL of citrate buffer (250 mM, pH 4.5), 10 μL of *p*-nitrophenyl-β-D-galactopyranoside (pNPGal; 4 mg/mL), and 100 μL of the enzyme source. After incubation at 37 °C for 30 min, the reaction was stopped by adding 1 mL of cold sodium carbonate buffer (500 mM, pH 10). The activity of β-galactosidase was estimated spectrophotometrically by reading the absorbance of the liberated *p*-nitrophenol at 405 nm (ε = 18,700). One unit (U) of β-galactosidase activity was defined as the amount of enzyme required for the hydrolysis of 1 μmol of substrate pNPGal per min, under the assay conditions.

*Growth on prebiotic oligosaccharides*

*E. durans* LAB18S cells were grown in BHI without shaking at 37 °C for 24 h. Then, the culture was inoculated (1%, v/v) in individual sterile vials containing M9 medium (5 g/L NH_4_Cl, 33.9 g/L Na_2_HPO_4_, 15 g/L KH_2_PO_4_, and 2.5 g/L NaCl), added with 10 g/L of either glucose, lactose, FOS or GOS and incubated at 37 °C. The growth was monitored by measuring the OD at 600 nm using spectrophotometer.

## Results

### Structure and general features of E. durans LAB18S genome

The genome sequence of *E. durans* LAB18S was obtained using the Illumina® MiSeq system, and compared with the complete genome sequence of *E. durans* KLDS6.0933 (GenBank accession number CP012366.1). The complete genome of *E. durans* LAB18S is composed of a chromosome with 2,867,357 bp, GC content of 38%, 2,579 CDSs, 108 RNAs and 180 pseudogenes (Table S1). By assembling the genome, a total of 82 contigs were obtained and a mean coverage of 31.7 x giving reliability to the results. Comparatively, the reference strain (*E. durans* KLDS6.0933) has 2,867,028 bp and the *E. durans* LAB18S genome is slightly larger with additional 329 bp.

The genes were grouped into subsystems through the RAST webservice (Figure S1). In brief, there are 126 genes for cell wall and capsule; 342 genes for carbohydrate transport and metabolism, which contains 17 genes related with fructooligosaccharides (FOS) and raffinose utilization; 63 genes for virulence, disease and defense, which contains adhesion, bacteriocins, resistance to antibiotics and toxic compounds, invasion and intracellular resistance genes; 2 genes for phages and prophages; 58 for membrane transport; 219 for protein metabolism; 6 for dormancy and sporulation, and 69 for stress response.

### Genes associated with probiotics properties

**Figure 1 -:**
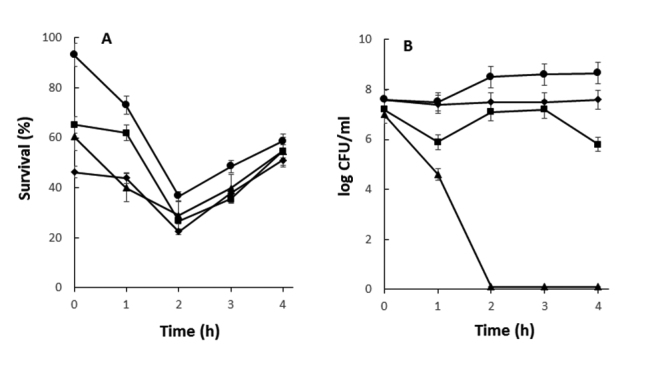
Tolerance of *E. durans* LAB18S to bile salts and acid pH. (A) The strain was incubated for up to 4 h at 37 °C in the presence of bile salts at concentrations 2.5 mg/mL (●), 5.0 mg/mL (■), 10 mg/mL (▲) or 15 mg/mL (♦). Results are expressed as percentage of surviving cells in comparison to incubation without bile salts used as a control. (B) The strain was incubated for up to 4 h at 37 °C in pH 2 (▲), 3 (■), 4 (♦) or 7 (●). Viable cell counts were monitored at each 1 h interval. Values are the means ± standard deviations of three independent experiments.

The *E. durans* LAB18S genome showed several genes that may be related with probiotic activity (Table 2). It encodes an S-layer protein (LIU RS11695), and two fibronectin-binding proteins (LIURS07910 and LIU RS10480), which may contribute to bacterial adherence. Besides, this genome carries an exopolysaccharide (EPS) cluster that could be related with improved adhesion properties and persistence in the gut. In addition, it also contains genes that can be associated to viability at lower pH (Na^+^/H^+^ antiporters) and bile salt tolerance (Table 2). In this regard, *E. durans* LAB18S demonstrated ability to survive at pH 3.0 and higher, and up to 15 mg/mL bile salts (Figure 1).

**Table 2 -:** Genes associated with potential probiotic properties of *E. durans* LAB18S.

Protein	Gene	Function
*Maintenance in the gastrointestinal tract*		
S-layer protein	*lbs*	Improves adhesion properties and persistence in the gut
Fibronectin-binding protein	*prtF*	Improves adhesion properties and persistence in the gut
Heat-shock protein 33	*hsp33*	Improves persistence in the gut
EPS cluster	*epsABCDE*	Improves adhesion properties and persistence in the gut
Na^+^/H^+^ antiporter	*nhaC*	Improves viability at low pH
Cyclopropane-fatty-acyl-phospholipid synthase	*Cfa*	Key protein in bile salt tolerance
*Bacteriocins and toxin-antitoxins*		
Microcin cluster	*micJ25*	Low molecular mass bacteriocins produced under stress conditions
Enterocin A immunity protein	*entI*	Putative protection against the effect of bacteriocin enterocin A
Colicin V precursor	*cvaC*	Kills sensitive cells by disrupting their membrane potential
Zeta-toxin	*pSM19035*	Inhibits cell wall biosynthesis
Toxin RelE	*relE*	Cleaves translating mRNA in the ribossomal A-site upon aminoacid starvation
*Resistance to heavy metals*		
Multi-copper oxidase	*cueO*	Provides copper tolerance
Copper-transporting efflux system	*cusCFBA*	Mediates resistance to copper and silver
Cation efflux system protein CzcA	*czcA*	Provides resistance to cobalt, zinc and cadmium
Mercuric reductase	*merA*	Provides resistance to mercury
*Carbohydrate utilization*		
Raffinose operon regulatory protein	*rafR*	Metabolism of fructooligosaccharides (FOS) and raffinose
Lactose operon	*lacZYA*	Metabolism of lactose and galactose
Maltodextrin phosphorylase	*malP*	Metabolism of maltodextrin and a-1,4-glucans
4-alpha-glucanotransferase	*malQ*	Starch metabolism

**Figure 2 -:**
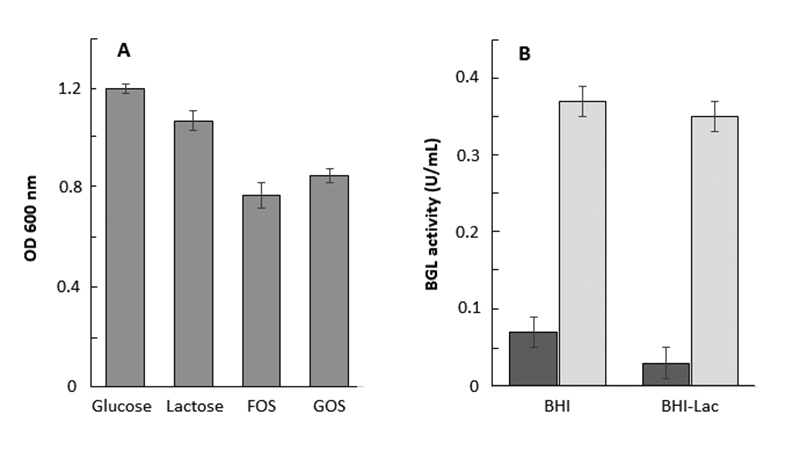
Phenotypical characteristics of *E. durans* LAB18S. (A) Growth on different carbon sources. The strain was cultivated in medium containing 10 g/L glucose, lactose, FOS or GOS and the cell density was estimated by OD_600_ after 24 h at 37^o^C. (B) The β-galactosidase (BGL) activity was measured in the cell culture supernatant (dark gray bars) and cell lysates (pale gray bars) after growth in BHI or BHI supplemented with 10 g/L lactose (BHI-Lac). Values are the means ± standard deviations of three independent experiments.

The potential for carbohydrate utilization was also analyzed and genes for fructooligosaccharide (FOS) and disaccharides utilization were found. Besides, the β-galactosidase (BGL) gene was identified in the genome (Table 2). These properties were confirmed by phenotypical assays showing the *E. durans* LAB18S has ability to growth on probiotic oligosaccharides FOS and GOS and produce BGL activity (Figure 2).

Secondary metabolite analysis revealed the presence of genes associated with colicin V, enterocin A, and the small bacteriocin microcin J25 (Table 2). In agreement, the culture supernatant of *E. durans* LAB18S showed inhibitory haloes ranging 9-10 mm against strains of *Listeria* spp.. Furthermore, two genes of toxin-antitoxin proteins, namely RelE and Zeta-toxin, were also identified. The BLAST algorithm was used to align the deduced colicin V sequence of *E. durans* LAB18S with colicin V and colicin V production protein CvpA from other genera and species. This sequence is quite conserved among different species of *Enterococcus*, *Bacillus* and *Carnobacterium* and strain *E. durans* LAB18S (Figure S2).

### Genes related to selenoproteins

The *E. durans* LAB18S genome contains seven genes involved in selenium metabolism (Table 3). Five genes encode typical selenoproteins, namely glutathione peroxidase (*gpx*), thioredoxin reductase (*trxB1, trxB2*), glycine reductase complex selenoprotein B (*grdB*), and peroxiredoxin (*prX*). Another two genes are related with selenium metabolism: L-seryl-tRNA selenium transferase (*selA*) and YggS family pyridoxal phosphate (*yggS*).

**Table 3 -:** Selenoprotein related genes predicted in *E. durans* LAB18S genome.

Protein	Gene	Function
*Selenoproteins*		
Glutathione peroxidase	*gpx*	Catalyzes the reduction of H_2_O_2_; protection against oxidative stress
Thioredoxin reductase	*trxB1*, *trxB2*	NADPH-depended oxidoreductase activity
Glycine reductase complex	*grdB*	Active protein in the peroxidase reaction
Peroxiredoxin	*prX*	Antioxidant enzyme that uses thioredoxin (Trx) to recharge after reducing H_2_O_2_
*Other selenium-related proteins*		
L-seryl-tRNA selenium transferase	*selA*	Converts seryl-tRNA(Sec) to selenocysteinyl-tRNA (Sec) required for selenoprotein biosynthesis
Selenocysteine-specific elongation factor	*selB*	Translation factor necessary for the incorporation of selenocysteine into proteins
YggS family pyridoxal phosphate	*yggS*	Decomposes selenocysteine to alanine and elemental Se or H_2_Se during selenium metabolism

In the analysis of the selenocysteine insertion sequence (SECIS) element, 1,274 hits were identified as candidates of bacterial SECIS (bSECIS)-like elements. These hits were divided into homologs of previously known selenoproteins (40 sequences) and candidates of selenoproteins (1,234 sequences). Then, optimal bSECIS elements and their predicted putative coding genes were identified as 26 known selenoproteins and 765 unknown bSECIS elements were detected. After the BLAST search was performed to filter out false positives, 12 bSECIS elements involved with selenium were identified. This indicates the ability of the *E. durans* LAB18S to produce selenoproteins.

After the BLAST search to filter out false positives, 12 bSECIS elements involved with selenium were identified. This indicates the ability of *E. durans* LAB18S to produce selenoproteins.

### Comparative analysis

**Figure 3 -:**
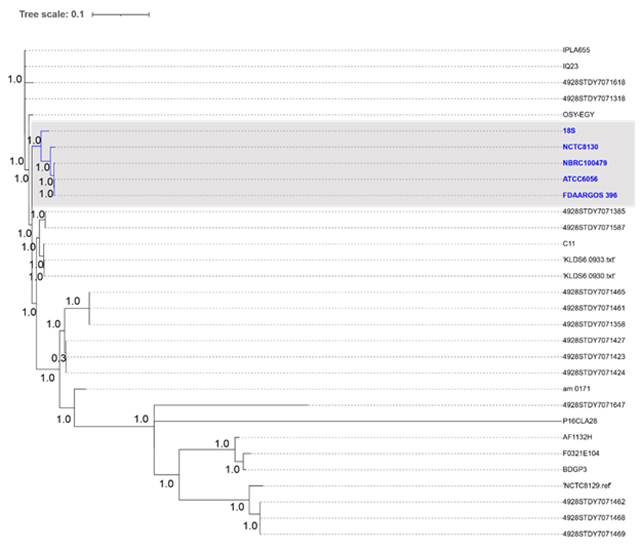
Core genome SNP tree of 31 *E. durans*. The phylogenomic reconstruction was built using Parsnp and Fast tree 2. The percentage of the reference that is covered by core alignments was above 60%. Strains related with *E. durans* LAB18S are highlighted in the grey box.

Virulence genes were not found in the *E. durans* genomes compared in this study, such as aggregation substance (*agg*), surface adhesins (*esp*, *ace*), sex pheromones (*cob*, *cpd*, *ccf*), D-alanylation of lipoteichoic acid (*dlt*), the lytic enzymes gelatinase (*gelE*) and hyaluronidase (*hyl*), and the toxin cytolysin (*cylA*). Antimicrobial resistance was checked against the ResFinder database and genes associated with tetracycline resistance, namely *tet*(M) and *tet*(O)-like were found in seven genomes of *E. durans*, mostly from fecal origin (Table 4). Only three enterococci under study did not present any plasmids, including *E. durans* LAB18S. Besides the absence of plasmids, these three strains also showed no virulence and antimicrobial resistance genes. The 31 genomes of *E. durans* were clusterized into a phylogenetic tree (Figure 3). *E. durans* LAB18S has been clusterized with isolates NCTC8130, FDAARGOS_396 and ATCC 6056, which are of fecal origin, and NRBC10079, which lacks source information. None of these isolates showed antimicrobial resistance or virulence genes. The presence of plasmids was found in these isolates, excepting for *E. durans* LAB18S (Table 4).

**Table 4 -:** Comparative analysis of the presence of virulence genes, antimicrobial resistance genes and plasmids of 31 *E. durans* genomes.

Species	Strain	Origin	Resistance		Virulence	Plasmids						
			*tet*(M)	*tet*(O)		*rep*1	*rep*2	*rep*4	*rep*11	*rep*18	*rep*US1	*rep*US15
*Enterococcus durans*	NCTC8129	Unknown										
*Enterococcus durans*	NCTC8130	Unknown										
*Enterococcus durans*	P16CLA28	Cloaca (*Gallus gallus*)										
*Enterococcus durans*	F0321E104	Feces (*Bos taurus*)										
***Enterococcus durans***	**LAB18S**	Frescal cheese										
*Enterococcus durans*	KLDS6.0930	Water										
*Enterococcus durans*	KLDS6.0933	Water										
*Enterococcus durans*	IQ23	Cheese										
*Enterococcus durans*	AF1132H	Feces (*Homo sapiens*)										
*Enterococcus durans*	ATCC6056	Feces (*Homo sapiens*)										
*Enterococcus durans*	IPLA655	Cheese										
*Enterococcus durans*	C11	Kimchi										
*Enterococcus durans*	OSY-EGY	Egyptian hard Cheese										
*Enterococcus durans*	am_0171	Feces (*Homo sapiens*)										
***Enterococcus durans***	**BDGP3**	Feces (*Drosophila melanogaster*)										
*Enterococcus durans*	4928STDY7071618	Feces (*Homo sapiens*)										
*Enterococcus durans*	4928STDY7071587	Feces (*Homo sapiens*)										
*Enterococcus durans*	4928STDY7071465	Feces (*Homo sapiens*)										
*Enterococcus durans*	4928STDY7071468	Feces (*Homo sapiens*)										
***Enterococcus durans***	**4928STDY7071461**	Feces (*Homo sapiens*)										
*Enterococcus durans*	4928STDY7071424	Feces (*Homo sapiens*)										
*Enterococcus durans*	4928STDY7071423	Feces (*Homo sapiens*)										
*Enterococcus durans*	4928STDY7071358	Feces (*Homo sapiens*)										
*Enterococcus durans*	4928STDY7071318	Feces (*Homo sapiens*)										
*Enterococcus durans*	4928STDY7071647	Feces (*Homo sapiens*)										
*Enterococcus durans*	4928STDY7071469	Feces (*Homo sapiens*)										
*Enterococcus durans*	4928STDY7071427	Feces (*Homo sapiens*)										
*Enterococcus durans*	4928STDY7071462	Feces (*Homo sapiens*)										
*Enterococcus durans*	4928STDY7071385	Feces (*Homo sapiens*)										
*Enterococcus durans*	FDAARGOS_396	Feces (*Homo sapiens*)										
*Enterococcus durans*	NBRC 100479	Unkwnown										

Black boxes indicate the presence of resistance genes, dark gray boxes indicate the presence of plasmids, light gray lines indicate the strains that were negative for virulence genes, antimicrobial resistance genes and plasmids.

## Discussion

Complete knowledge of genome sequences may allow a precise genetic analysis of probiotic bacteria. This includes the genetic features that can be associated with beneficial effects and those potentially associated with undesirable characteristics. The genus *Enterococcus* contains strains associated with severe infections, while other strains form part of the commensal human microbiome of the mouth, skin, and gut. Some strains have probiotic properties, including *E. durans* (Liaskovs’kyĭ et al., 2008; Li *et al*., 2018). Interestingly, virulence is very different among enterococci derived from community or hospital environments, which appears to be associated to some strain-specific genetic features (Douillard and de Vos, 2014). Thus, the study of whole genomes is relevant to acquire information on the potential benefits and drawbacks. In this work, the genome of *E. durans* LAB18S isolated from Minas Frescal cheese showed some desirable characteristics for a probiotic strain.

The survival of probiotic bacteria under gastrointestinal tract conditions has been extensively studied. Probiotics, after ingestion, are exposed to the acidic conditions and the activity of digestive enzymes of the stomach. *E. durans* LAB18S is equipped with a gene coding for Na^+^/H^+^ antiporter, contributing to regulate intracellular pH (Guo et al., 2015). The reduction of bacterial survival in the gut may be due to secretion of bile that breaks the microbial cell membrane, and tolerance to bile salt concentrations between 0.15 and 0.5% has been recommended for probiotics (Lavermicocca et al., 2008). The gene encoding cyclopropane-fatty-acyl-phospholipid synthase (HUO 05315), present in the genome of *E. durans* LAB18S, might be associated with bile salt tolerance. Comparative proteomic studies on *Lactobacillus plantarum* identified this enzyme as a key protein in bile tolerance (Hamon et al., 2011). Bile salt hydrolase identified in the genome of *E. durans* KLDS6.0933, has been also associated with cholesterol removal ability (Li et al., 2018).

Adhesive properties can prolong the contact between bacteria and the host and therefore enhance the desired probiotic effect (Botta et al., 2014). Although mucus-binding proteins and adhesion genes are absent, *E. durans* LAB18S genome presents an S-layer protein (LIURS 11695), and fibronectin-binding proteins (LIURS 07910 and LIURS 10480), which may contribute to bacterial adherence. In addition, a gene encoded aggregation-promoting factor (LIURS 03835) was also identified, suggesting that this strain can bind to receptors in the gut environment (Senan et al., 2015). Some EPS produced by probiotics can improve its adhesion properties and its persistence in the gut (Ruas-Madiedo et al., 2006), and the *E. durans* LAB18S genome carries an EPS cluster. All these genetic elements corroborate to the potential adhesive characteristics of *E. durans* LAB18S.

The production of bacteriocins by probiotic strains has been recognized as a desirable feature (Hegarty et al., 2016). Analysis for secondary metabolite clusters of *E. durans* LAB18S genome revealed the presence of genes associated with the synthesis of microcin J25, colicin V and enterocin A, which may endow competitive advantages to combat pathogenic bacteria. The inhibitory activity of *E. durans* LAB18S against *Listeria* spp. agrees with the typical antilisterial activity of *Enterococcus* bacteriocins (Rocha et al., 2019). Colicin V is produced by many strains of *Escherichia coli* and its precursor peptide is similar to some bacteriocins of the *Enterobacteriaceae* family, which fits the definition of class II bacteriocins from Gram-positive bacteria (Håvarstein et al., 1994). The transfer of genes encoding bacteriocins from Gram-negative bacteria, such as colicin V, to food-grade lactic acid bacteria (LAB) host has been described (Langa et al., 2017). In this regard, Horn et al. (2004) were the first to show the coproduction of nisin and colicin V in *Lactococcus lactis* as a host enhancing the antimicrobial activity against both Gram-positive and Gram-negative bacteria.

The *E. durans* LAB18S genome contains genes of toxin-antitoxin systems, which have been associated with survival under stress conditions (Fernández-García et al., 2016). Zeta-toxin is bactericidal for *Bacillus subtilis* and bacteriostatic for *E. coli*, while the toxin RelE degrades mRNA at specific sequences when it is bound to the ribosomal A site (Pedersen et al., 2003). As a concern, the presence of omega/epsilon/zeta toxin-antitoxin system seems to stabilize plasmids carrying *vanA* in *E. faecium* and *E. faecalis* resistant to vancomycin (Fernández-Gracía *et al*., 2016).

Genes related to the metabolism of molecules associated with prebiotic properties were also identified. The strain LAB18S presented genes related to the use of frutooligosaccharides (FOS), a non-digestible dietary component that undergo selective colonic fermentation. FOS cause significant changes in the composition of the gut microbiota, increasing the numbers of potentially health-promoting bacteria and reducing potentially harmful species, respectively (Slavin, 2013). Cultivation of *E. durans* LAB18S on FOS revealed an increased number of overexpressed proteins, including L-asparaginase and arginine deiminase, two enzymes of clinical importance for the treatment of cancer (Comerlato et al., 2020). The BGL gene was also detected in the genome. This enzyme is produced by several LAB with both hydrolase and transglycosylase activities, beneficial from technological and health point of views for applications as probiotic cultures in dairy industry or synthesis of prebiotic GOS (Meira et al., 2012). Because they are not digested by humans, GOS represents a rich source of substrate for probiotic organisms, including *Enterococcus* (Park and Oh, 2010).

Selenium is an essential metalloid required for the expression of selenoproteins. It was previously observed that *E. durans* LAB18S bioaccumulates selenium when grown in medium containing Na_2_SeO_3_ (Pieniz et al., 2017). Selenium was mainly found as selenoproteins, reaching 2.6 mg/g biomass. Selenoprotein genes, to insert SEC into UGA codons, have developed a stem-loop shaped RNA structure, called SECIS. These SECIS elements are located downstream of the Sec UGA codons in bacteria. Through a computer program we were able to identify conserved structural characteristics of these structures. Bacterial SECISearch recognize a bacterial consensus SECIS element in sequence databases and the results indicate the ability of the *E. durans* LAB18S to produce selenoproteins. Selenium antioxidant properties stimulates the activity of some antioxidant enzymes, such as glutathione peroxidase, thioredixin reductase, and iodothyronine deiodinase, which contain selenocysteine (Lin et al., 2015). One biological form of Se has been identified as selenocysteine (Sec) (Hatfield and Gladyshev, 2002), but selenium could form selenomethionine (SeMet) by replacing sulfur in methionine and thus could be incorporated into proteins instead of methionine (Schrauzer, 2000). Although some microorganisms are capable of transforming high concentrations of selenium into selenate and selenite, only few studies on selenite uptake and biotransformation have been conducted with probiotic microorganisms (Zhang et al., 2009; Pieniz *et al*., 2017). Comparative genomic analyses were performed in order to identify new genes associated to Se utilization in *Enterococcus faecalis*. Seven candidate genes for selenoproteins were identified (Zhang *et al*., 2008), the same number found in this study.

Enterococci may have resistance to various antibiotics, due to their innate resistance to widely used antibiotics such as penicillin or to their ability to easily acquire antimicrobial resistance, especially by horizontal gene transfer. Horizontal transfer of antimicrobial resistance in enterococci has been associated with mobile genetic elements, such as plasmids and transposons (Palmer et al., 2012; Beukers et al., 2015). Resistance to tetracycline in *Enterococcus* spp. is frequently associated with the resistance genes *tet*(M) and *tet*(O) (Roberts, 2005; Anderson et al., 2016). Recently, a PCR-based plasmid classification system has been established by targeting specific replicon initiation genes (*rep*) of plasmid DNA. Rep-family, already found in the genus *Enterococcus*, may confer multiple antibiotic resistance as well as the mechanism of stabilization of toxin-antitoxin plasmids (Zankari et al., 2012; Bonacina et al., 2017). The absence of such genetic elements in *E. durans* LAB18S reinforce its promising as probiotic strain. Another recent study concludes that a cheese isolate *E. faecalis* does not represent a substantial reservoir of antimicrobial resistance and virulence when compared to clinical strains (Silvetti et al., 2019). The *E. durans* LAB18S genome was more closely to human feces genomes, which can be explained in part because enterococci are enteric bacteria commonly associated with the gastrointestinal tract of animals. In this regard, many probiotic lineages have been identified from animals or human feces (Hanchi et al., 2018; Nagpal et al., 2018; Bazireh et al., 2020).

In summary, the genome of *E. durans* LAB18S presents a variety of genes that can be associated with probiotic properties, such as adhesion properties, viability at lower pH, bile salt tolerance, production of bacteriocins, and utilization of prebiotic molecules. Besides, this strain presents genes encoding for known selenoproteins, which should contribute to the antioxidant properties. In comparison with other *E. durans* genomes, *E. durans* LAB18S was the only food isolate with absence of plasmids, virulence and antimicrobial resistance genes. *E. durans* LAB18S exhibited a probiotic potential and its potential health benefit and application as probiotic strain in the feed industry merits future investigation. This work significantly improved the knowledge on the genetic characteristics of this promising strain.

## Supplementary material

The following online material is available for this article:

Table S1 - General genome features of *E. durans* LAB18S compared with *E. durans* KLDS6.0933.

Figure S1 -
*E. durans* LAB18S genes grouped into subsystems by RAST.

Figure S2 - Multiple sequence alignment of colicin V gene from *E. durans* LAB18S.

## References

[B1] Anderson AC, Jonas D, Huber I, Karygianni L, Wölber J, Hellwig E, Arweiler N, Vach K, Wittmer A, Al-Ahmad A (2016). Enterococcus faecalis from food, clinical specimens, and oral sites: Prevalence of virulence factors in association with biofilm formation. Front Microbiol.

[B2] Bankevich A, Nurk S, Antipov D, Gurevich AA, Dvorkin M, Kulikov AS, Lesin VM, Nikolenko SI, Pham SON, Prjibelski AD (2012). SPAdes: A new genome assembly algorithm and its applications to single-cell sequencing. J Comput Biol.

[B3] Bazireh H, Shariati P, Azimzadeh Jamalkandi S, Ahmadi A, Boroumand MA (2020). Isolation of novel probiotic Lactobacillus and Enterococcus strains from human salivary and fecal sources. Front Microbiol.

[B4] Beukers AG, Zaheer R, Cook SR, Stanford K, Chaves AV, Ward MP, McAllister TA (2015). Effect of in-feed administration and withdrawal of tylosin phosphate on antibiotic resistance in enterococci isolated from feedlot steers. Front Microbiol.

[B5] Bonacina J, Suárez N, Hormigo R, Fadda S, Lechner M, Saavedra L (2017). A genomic view of food-related and probiotic Enterococcus strains. DNA Res.

[B6] Botta C, Langerholc T, Cencic A, Cocolin L (2014). In vitro selection and characterization of new probiotic candidates from table olive microbiota. PLoS One.

[B7] Byappanahalli MN, Nevers MB, Korajkic A, Stanley ZR, Harwood VJ (2012). Enterococci in the environment. Microbiol Mol Biol Rev.

[B8] Carattoli A, Zankari E, García-Fernández A, Voldby LM, Lund O, Villa L, Aarestrup FM, Hasman H (2014). In silico detection and typing of plasmids using PlasmidFinder and plasmid multilocus sequence typing. Antimicrob Agents Chemother.

[B9] Clemente JC, Ursell LK, Partry LW, Knight R (2012). The impact of the gut microbiota on human health: An integrative view. Cell.

[B10] Comerlato CB, Zhang X, Walker K, Brandelli A, Figeys D (2020). Comparative proteomic analysis reveals metabolic variability of probiotic Enterococcus durans during aerobic and anaerobic cultivation. J Proteomics.

[B11] de Man JC, Rogosa M, Sharpe ME (1960). Medium for the cultivation of lactobacilli. J Appl Bacteriol.

[B12] Douillard FP, de Vos WM (2014). Functional genomics of lactic acid bacteria: From food to health. Microb Cell Fact.

[B13] Erkkila S, Petaja E (2000). Screening of commercial meat starter cultures at low pH and in the presence of bile salts for potential probiotic use. Meat Sci.

[B14] Fernández-García L, Blasco L, Lopez M, Bou G, García-Contreras R, Wood T, Tomas M (2016). Toxin-antitoxin systems in clinical pathogens. Toxins (Basel).

[B15] Franz CM, Huch M, Abriouel H, Holzapfel W, Gálvez A (2011). Enterococci as probiotics and their implications in food safety. Int J Food Microbiol.

[B16] Galano E, Mangiapane E, Bianga J, Palmese A, Pessione E, Szpunar J, Lobinski R, Amoresano A (2013). Privileged incorporation of selenium as selenocysteine in Lactobacillus reuteri proteins demonstrated by selenium-specific images and proteomics. Mol Cell Proteomics.

[B17] Gilliland SE, Staley TE, Bush LJ (1984). Importance of bile tolerance of Lactobacillus acidophilus used as dietary adjunct. J Dairy Sci.

[B18] Guo L, Li T, Tang Y, Yang L, Huo G (2015). Probiotic properties of Enterococcus strains isolated from traditional naturally fermented cream in China. Microb Biotechnol.

[B19] Hamon E, Horvatovich P, Izquierdo E, Bringel F, Marchioni E, Aoudé-Werner D, Ennahar S (2011). Comparative proteomic analysis of Lactobacillus plantarum for the identification of key proteins in bile tolerance. BMC Microbiol.

[B20] Hanchi H, Mottawea W, Sebei K, Hammami R (2018). The genus Enterococcus: between probiotic potential and safety concerns - An update. Front Microbiol.

[B21] Hang YD, Woodams EE (1994). Apple pomace: A potential substrate for production of β-glucosidase by Aspergillus foetidus. LWT Food Sci Technol.

[B22] Hatfield DL, Berry MJ, Gladyshev VN (2006). Selenium - Its molecular biology and role in human health.

[B23] Hatfield DL, Gladyshev VN (2002). How selenium has altered our understanding of the genetic code. Mol Cell. Biol.

[B24] Håvarstein LS, Holo H, Nes IF (1994). The leader peptide of colicin V shares consensus sequences with leader peptides that are common among peptide bacteriocins produces by Gram positive bacteria. Microbiology (Reading).

[B25] Hegarty JW, Guinane CM, Ross RP, Hill C, Cotter PD (2016). Bacteriocin production: A relatively unharnessed probiotic trait?. F1000Res.

[B26] Hill C, Guarner F, Reid G, Gibson GR, Merenstein DJ, Pot B, Morelli L, Canani RB, Flint HJ, Salminen S (2014). Expert consensus document: The International Scientific Association for Probiotics and Prebiotics (consensus statement on the scope and appropriate use of the term probiotic. Nat Rev Gastroenterol Hepatol.

[B27] Horn N, Fernandez A, Dodd HM, Gasson MJ, Rodriguez JM (2004). Nisin-controlled production of pediocin PA-1 and colicin V in nisin- and non-nisin-producing Lactococcus lactis strains. Appl Environ Microbiol.

[B28] Joensen KG, Scheutz F, Lund O, Hasman H, Kaas RS, Nielsen EM, Aarestrup FM (2014). Real-time whole-genome sequencing for routine typing, surveillance, and outbreak detection of verotoxigenic Escherichia coli. J Clin Microbiol.

[B29] Ladero V, Linares DM, del Rio B, Fernandez M, Martin MC, Alvarez MA (2013). Draft genome sequence of the tyramine producer Enterococcus durans strain IPLA 655. Genome Announc.

[B30] Langa S, Arqués J, Medina M, Landete J (2017). Coproduction of colicin V and lactic acid bacteria bacteriocins in lactococci and enterococci strains of biotechnological interest. J Appl Microbiol.

[B31] Lavermicocca P, Valerio F, Lonigro SL, Di Leo A, Visconti A (2008). Antagonistic activity of potential probiotic lactobacilli against the ureolytic pathogen Yersinia enterocolitica. Curr Microbiol.

[B32] Letunic I, Bork P (2019). Interactive Tree Of Life (iTOL) v4: Recent updates and new developments. Nucleic Acids Res.

[B33] Li B, Evivie SE, Jin D, Meng Y, Li N, Yan F, Huo G, Liu F (2018). Complete genome sequence of Enterococcus durans KLDS6.0933, a potential probiotic strain with high cholesterol removal ability. Gut Pathog.

[B34] Liaskovs’kyĭ TM, Pidhors’kyĭ VS, Kovalenko NK, Harmasheva IL, Muchnyk FV (2008). Identification of probiotic lactic acid bacteria strains. Mikrobiol Z.

[B35] Lin HC, Ho SC, Chen YY, Khoo KH, Hsu PH, Yen HCS (2015). CRL2 aids elimination of truncated selenoproteins produced by failed UGA/Sec decoding. Science.

[B36] Martín R, Langella P (2019). Emerging health concepts in the probiotics field: Streamlining the definitions. Front Microbiol.

[B37] Medema MH, Blin K, Cimermancic P, de Jager V, Zakrzewski P, Fischbach MA, Weber T, Takano E, Breitling R (2011). antiSMASH: Rapid identification, annotation and analysis of secondary metabolite biosynthesis gene clusters in bacterial and fungal genome sequences. Nucleic Acids Res.

[B38] Meira SMM, Helfer VE, Velho RV, Lopes FC, Brandelli A (2012). Probiotic potential of Lactobacillus spp. isolated from Brazilian regional ovine cheeses. J Dairy Res.

[B40] Nagpal R, Wang S, Ahmadi S, Hayes J, Gagliano J, Subashchandrabose S, Kitzman DW, Becton T, Read R, Yadav H (2018). Human-origin probiotic cocktail increases short-chain fatty acid production via modulation of mice and human gut microbiome. Sci Rep.

[B41] Palmer KL, Godfrey P, Griggs A, Kos VN, Zucker J, Desjardins C, Cerqueira G, Gevers D, Walker S, Wortman J (2012). Comparative genomics of enterococci: variation in Enterococcus faecalis, clade structure in E. faecium, and defining characteristics of E. gallinarum and E. casseliflavus. mBio.

[B42] Park AR, Oh DK (2010). Galacto-oligosaccharide production using microbial beta-galactosidase: Current state and perspectives. Appl Microbiol Biotechnol.

[B43] Pedersen K, Zavialov AV, Pavlov M, Elf J, Gerdes K, Ehrenberg M (2003). The bacterial toxin RelE displays codon-specific cleavage of mRNAs in the ribosomal A site. Cell.

[B44] Pieniz S, Moura TM, Cassenego AP, Andreazza R, Frazzon AP, Oliveira FAO, Brandelli A (2015). Evaluation of resistance genes and virulence factors in a food isolated Enterococcus durans with potential probiotic effect. Food Control.

[B45] Pieniz S, Andreazza R, Mann MB, Camargo FAO, Brandelli A (2017). Bioaccumulation and distribution of selenium in Enterococcus durans. J Trace Elem Med Biol.

[B46] Price MN, Dehal PS, Arkin AP (2010). FastTree 2 - Approximately maximum-likelihood trees for large alignments. PLoS One.

[B47] Roberts MC (2005). Update on acquired tetracycline resistance genes. FEMS Microbiol Lett.

[B48] Rocha KR, Perini HF, Souza CM, Schueler J, Tosoni NF, Furlaneto MC, Furlaneto-Maia L (2019). Inhibitory effect of bacteriocins from enterococci on developing and preformed biofilms of Listeria monocytogenes, Listeria inovanii and Listeria innocua. World J Microbiol Biotechnol.

[B49] Ruas-Madiedo P, Gueimonde M, Margolles A, de los Reyes-Gavilán CG, Salminen S (2006). Exopolysaccharides produced by probiotic strains modify the adhesion of probiotics and enteropathogens to human intestinal mucus. J Food Prot.

[B50] Schrauzer GN (2000). Selenomethionine: a review of its nutritional significance, metabolism and toxicity. J Nutr.

[B51] Senan S, Prajapati JB, Joshi CG (2015). Whole-genome based validation of the adaptive properties of Indian origin probiotic Lactobacillus helveticus MTCC5463. J Sci Food Agric.

[B52] Silvetti T, Morandi S, Brasca M (2019). Does Enterococcus faecalis from traditional raw milk cheeses serve as a reservoir of antibiotic resistance and pathogenic traits?. Foodborne Pathog Dis.

[B53] Slavin J (2013). Fiber and prebiotics: Mechanisms and health benefits. Nutrients.

[B54] Treangen TJ, Ondov BD, Koren S, Phillippy AM (2014). The Harvest suite for rapid core-genome alignment and visualization of thousands of intraspecific microbial genomes. Genome Biol.

[B55] Zankari E, Hasman H, Cosentino S, Vestergaard M, Rasmussen S, Lund O, Aarestrup FM, Larsen MV (2012). Identification of acquired antimicrobial resistance genes. J Antimicrob Chemother.

[B56] Zhang Y, Gladyshev VN (2005). An algorithm for identification of bacterial selenocysteine insertion sequence elements and selenoprotein genes. Bioinformatics.

[B57] Zhang Y, Turanov AA, Hatfield DL, Gladyshev VN (2008). In silico identification of genes involved in selenium metabolism: Evidence for a third selenium utilization trait. BMC Genomics.

[B58] Zhang B, Zhou K, Zhang J, Chen Q, Liu G, Shang N, Qin W, Li P, Lin F (2009). Accumulation and species distribution of selenium in Se-enriched bacterial cells of the Bifidobacterium animalis 01. Food Chem.

[B59] Zhong Z, Zhang W, Song Y, Liu W, Xu H, Xi X, Menghe B, Zhang H, Sun Z (2017). Comparative genomic analysis of the genus Enterococcus. Microbiol Res.

