# Supplementary material for: Genomic analysis of Enterococcus durans LAB18S, a potential probiotic strain isolated from cheese
Source: Genet Mol Biol. 2022 Feb 25;45(1):e20210201. doi: 10.1590/1678-4685-GMB-2021-0201 (PMC8894896; doi:10.1590/1678-4685-GMB-2021-0201)
Supplement: Table S1 - [file 1415-4757-GMB-45-1-e20210201-s1.pdf]

Genetics and Molecular Biology
DOI: https://doi.org/10.1590/1678-4685-GMB-2021-0201

Supplementary Material to "Genomic analysis of *Enterococcus durans* LAB18S, a potential probiotic strain isolated from cheese"

Table S1 - General genome features of *E. durans* LAB18S compared with *E. durans* KLDS6.0933.

**Feature**	***E. durans* LAB18S**	***E. durans* KLDS6.0933**
Size (bp)	2,867,357	2,867,028
GC content (%)	38.1	37.8
Predicted genes	2669	2737
Protein coding genes (CDS)	2579	2333
Pseudogenes	180	323
rRNA	36	18
tRNA	68	68
ncRNA	4	4
