# Supplementary material for: Genomic analysis of Enterococcus durans LAB18S, a potential probiotic strain isolated from cheese
Source: Genet Mol Biol. 2022 Feb 25;45(1):e20210201. doi: 10.1590/1678-4685-GMB-2021-0201 (PMC8894896; doi:10.1590/1678-4685-GMB-2021-0201)
Supplement: Figure S1 - [file 1415-4757-GMB-45-1-e20210201-s2.pdf]

Genetics and Molecular Biology
DOI: https://doi.org/10.1590/1678-4685-GMB-2021-0201

Supplementary Material to “Genomic analysis of *Enterococcus durans* LAB18S, a potential probiotic strain isolated from cheese”

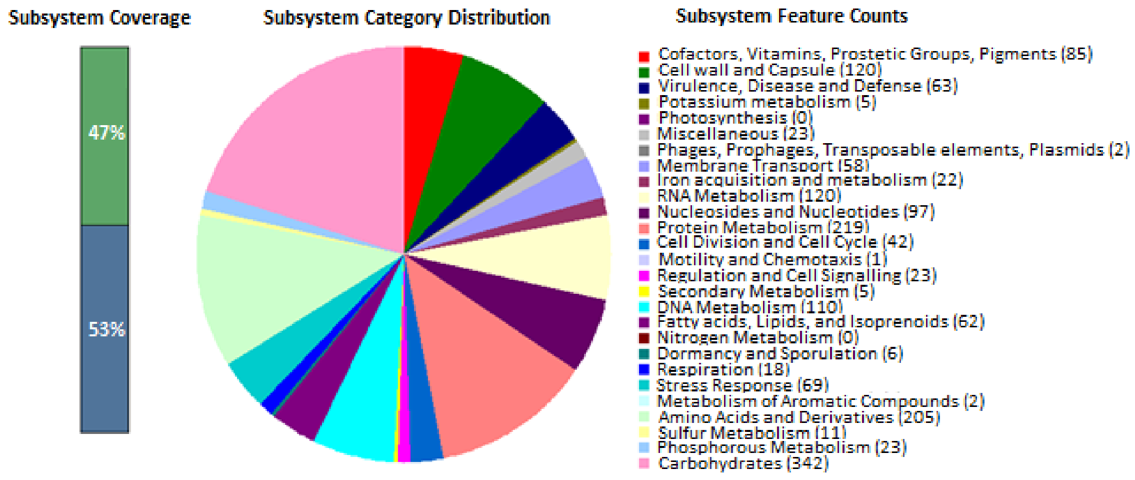

Figure S1 - *E. durans* LAB18S genes grouped into subsystems by RAST.
