# Supplementary material for: Genomic analysis of Enterococcus durans LAB18S, a potential probiotic strain isolated from cheese
Source: Genet Mol Biol. 2022 Feb 25;45(1):e20210201. doi: 10.1590/1678-4685-GMB-2021-0201 (PMC8894896; doi:10.1590/1678-4685-GMB-2021-0201)
Supplement: Figure S2 - [file 1415-4757-GMB-45-1-e20210201-s3.pdf]

Genetics and Molecular Biology
DOI: https://doi.org/10.1590/1678-4685-GMB-2021-0201

Supplementary Material to "Genomic analysis of *Enterococcus durans* LAB18S, a potential probiotic strain isolated from cheese"

```
Col V, E. durans LAB18S      1   MLSLLILFILLIAFLSGARRGFAMQVVYTIGYVLSFFAAQHFYKQLADHLELYIPYPAVTSTSKMVFFDQAISFRLDEA   79
Col V, E. durans             1   MLSLLILFILLIAFFSGARRGFAMQVVYTIGYVLSFFAAQHFYKQLADHLELYIPYPAVTSTSKMVFFDQAISFRLDEA   79
CvpA Family, E. hirae        1   MLSLLILFILLLAFFSGARRGFSMQVVYTIGYILSFMAAQHFYKDLASRLELYIPYPAVTSNSQMVFFDQTFSFRLDEA   79
CvpA Family, Bacilli         1   MLSLLIIIFLLIAFFSGAKRGFALQVVYTVGYLVSFLVAQHFYKQLANHLELYIPYPAVTPTSDLVFFDQTISFSLDQA   79
CvpA Family E. mundtii       1   MLSLLILFILLIAFFSGASRGFALQGIYLIGYFVSFLAAQTYYKTLASHLQLYIPYPAVTANSNLVFFDQAFSFKLDEA   79
CvpA Family E. faecalis      1   MLTLLILFILLIAFFSGARRGFTMQAVFTVGYVISFIAAQHFYKPLANHLRLYIPYPAVTPDSQMAFFDQARSLSLDQA   79
Col V, E. faecium            1   MLSLLILFLLLIAFFSGARRGFALQIIFAIGYVLSFIAAQHFYKPLASHLELYIPYPAVTPTSKLAFFDQVFAFHLDEA   79
CvpA Family, C. inhibens     1 mLMTVIIVLLLAIGAYSGARRGLIILQLVLTIGYFISYLLAGKYYQTLGSHLELIVPYPSASESSQFVFYNQALGFDLDGA   80
CvpA Family, C. viridans     1 mLMTVLIVLILAMGAYSGARRGLVLQFVFTIGYFVSYLLARNYYQLLGSHLELIVPYPSATESSQFVFYDQALGFNLDGA   80

Col V, E. durans LAB18S     80 FYAGVAFLLILLAGALITRFIGIFAHSLTYVPVLRQVDWLAGGVLSVVVAYVTIFLLLSLLTLVPVDFIQNQFSGNSLAR 159
Col V, E. durans           80 FYAGVAFLLILLAGALITRFIGIFAHSLTYVPVLRQVDWLAGGVLSVVVAYVTIFLLLSLLTLVPVDFIQNQFSGNSLAR 159
CvpA Family, E. hirae      80 FYAGVAFLLILFIGGLLTRFIGIFVHSLTYIPILKQVDWLAGGILSLIVAYVTIFLLLSLLTFVPVDIVQKQFSGNSLAR 159
CvpA Family, Bacilli       80 FYAGIAFLLILMAGWLITRFVGIFVHSLTYLPVLKQVDWLAGGLLSVVVTFVMIFLLLSLLSFVPSDFIQNQFRSSGLAR 159
CvpA Family E. mundtii     80 FYAGVAFLIILFIGWLVTRFIGVFAHGLTFIPVLKQLDWVAGGILSVIITYISLFLVLRLLTFIPVGFIQNQFNGNNLAT 159
CvpA Family E. faecalis    80 FYAGVAFLAIFAAGWLITRFIGVFLHGLTYVPVLRQADWIAGGILSMVVAYAVIFMLLSLLMMVPLDSIQNLFKSNGLPR 159
Col V, E. faecium          80 FYAGTAFLIILLIGWLLTRFVGVFVHGLTYVPILRQVDWIAGGILSLIMAYVTIFLILQLLAFVPLD------------ 146
CvpA Family, C. inhibens   81 FYNGVAFILILFVGWLITRFVGGLLNSLTFIPVLKQLNALGGAILNVIVSYVAIFLVLFLLTMVPIDAIQEAFNTSWLAR 160
CvpA Family, C. viridans   81 FYNGVAFITILFVGWLITRFVGGLLNAVTLIPVIKQLNTLGGALLNVIVSYIAIFIVLFLLTMVPVDAIQESFNNSWLAR 160

Col V, E. durans LAB18S   160 FIVEKTPLLSDKIHDLWVTNIINq   183
Col V, E. durans         160 FIVEKTPLLSDKIHDLWVTNIINq   183
CvpA Family, E. hirae    160 FIVDQTPFLTNKIHDLWITNVIN-   182
CvpA Family, Bacilli     160 YIVENTPILTNKIYDLWITRVIG-   182
CvpA Family E. mundtii   160 FMVERTPILANKIYDLWVTQVIN-   182
CvpA Family E. faecalis  160 FIVENTPVLSNKIYDLWITRIIG-   182
Col V, E. faecium            ------------------------
CvpA Family, C. inhibens 161 TIVEDTPVISAQLYNLWIETSLK-   183
CvpA Family, C. viridans 161 TIVEDTPVISAQLYNWWIESSLK-   183
```

Figure S2 - Multiple sequence alignment of colicin V gene from *E. durans* LAB18S.
